# Esophageal stenosis secondary to severe loxoscelism: A case report

**DOI:** 10.1002/jpr3.70107

**Published:** 2025-12-17

**Authors:** Lucas Rocha Alvarenga, Mariana Di Paula Rodrigues, Marise Helena Cardoso Tofoli, Patrícia dos Santos Oliveira

**Affiliations:** ^1^ State University of Campinas – UNICAMP Campinas Brazil; ^2^ Federal University of Goias – UFG Goiânia Brazil

**Keywords:** esophageal strictures, *Loxosceles*, spider bites

## Abstract

Loxoscelism is a public health issue in tropical countries, particularly in Brazil. It can affect children of all ages and may lead to severe and irreversible injuries. We report the case of an infant who suffered a severe loxoscelism accident in the cervical region, progressing to esophageal stricture requiring multiple dilations. Fortunately, in this case, the outcome was favorable despite the need for surgical and endoscopic interventions. This case highlights the importance of both individual and collective preventive measures to reduce the incidence of such accidents, especially in the pediatric population.

## 1 INTRODUCTION

Loxoscelism represents a public health problem in Brazil. The accident is initially painless and may be underestimated or associated with another clinical entity. The toxin contains enzymes known to cause dermo‐necrosis (cutaneous form) and systemic manifestations such as hemolysis, thrombocytopenia, and renal failure (cutaneous‐visceral form).^1^, ^2^ The cutaneous form causing esophageal stenosis by contiguity is a rare presentation and unprecedented in the world literature.^3^, ^4^, ^5^

## 2 CASE REPORT

**Figure 1:**
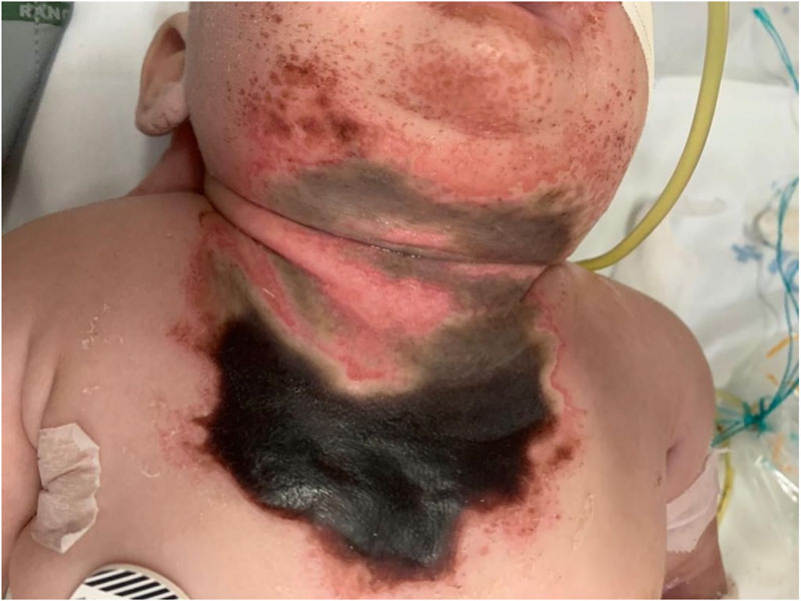
Extensive necrotic skin lesion in the anterior cervicothoracic region measuring 8.0 x 6.0 cm surrounded by petechiae.

A 3‐month‐old girl was admitted to the emergency room with a necrotic skin lesion in the anterior cervicothoracic region measuring 8.0 × 6.0 cm (Figure 1), with no defined etiology or chronology. The infant rapidly progressed to respiratory failure, coagulation disorder, and hypoglycemia. Based on the clinical presentation and local epidemiology, the infant received anti‐lonomia serum without clinical response.

**Figure 2:**
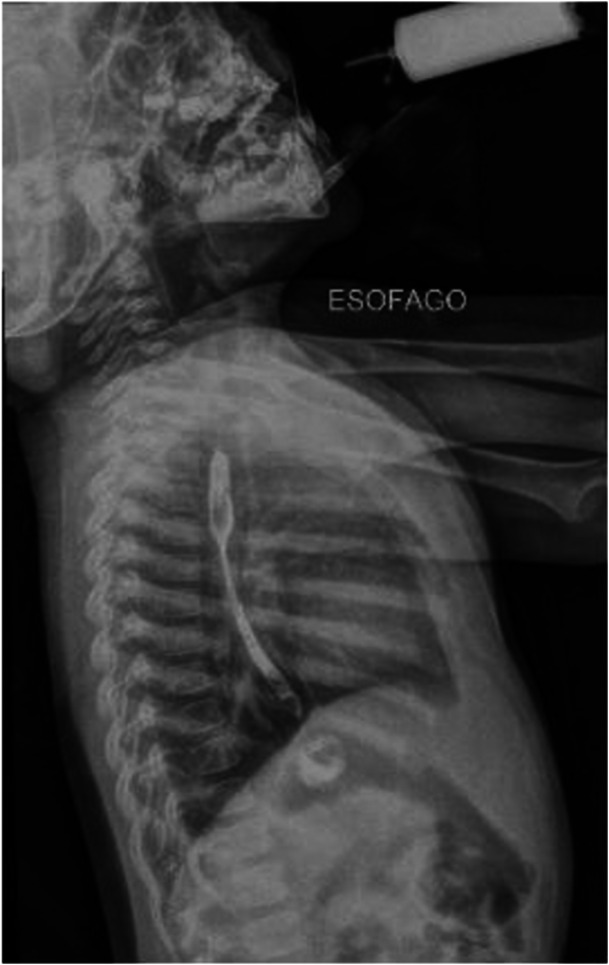
Contrast esophagogram showing narrowing in the middle third of the esophagus.

**Figure 3:**
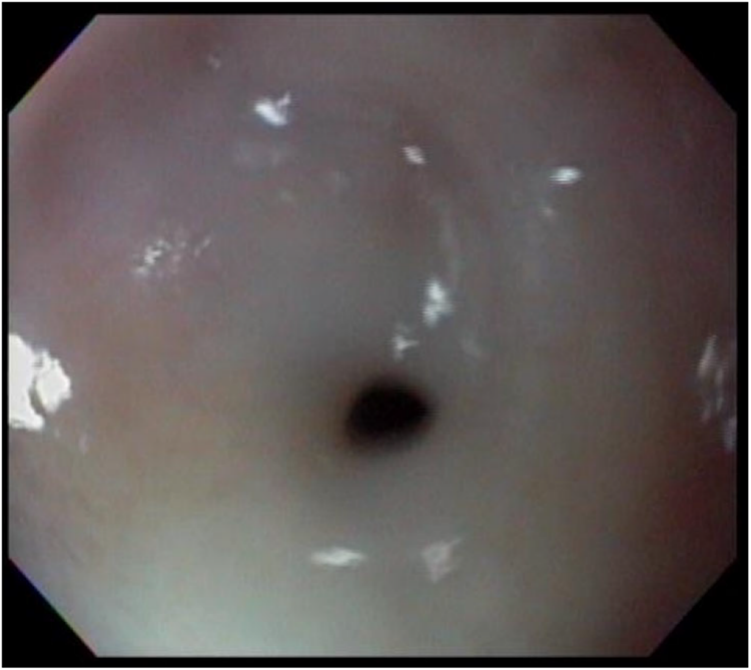
Upper digestive endoscopy showing stenosis between 10 and 15 cm of the upper dental arch.

After 48 h, a family member found a Loxosceles spider near the infant's crib and antiloxoscelic serum was started, which reversed the coagulation disorder and hypoglycemia. At 10 months of age, the patient began to vomit, sialorrhea, and food impaction. An esophagogram (Figure 2) revealed a narrowing in the middle third of the esophagus and the upper digestive endoscopy (Figure 3) detected stenosis between 10 and 15 cm of the upper dental arch. During the follow‐up, the patient required 21 esophageal dilations with Savary‐Gilliard probes 7, 9, and 11, with intervals between 2 and 4 weeks for 2 years. At present, the patient has been discharged following esophageal dilation procedures and is free from dysphagia. This is the only reported case to date of esophageal stenosis secondary to a loxoscelism accident, a public health problem in Brazil.

## 3 DISCUSSION

In Brazil, Loxosceles was the most common arachnid accident, with a higher prevalence in male adults between the third and sixth decades of life. In 2023, in Brazil, according to the DataSUS database, 8748 accidents by Loxosceles occurred, 1.1% of which were in children under 1 year of age. The cutaneous‐visceral clinical presentation, less frequent and with greater morbidity, represents only 20% of symptomatic cases and the cervicothoracic location is uncommon, even in the pediatric age group.

## 4 CONCLUSION

Esophageal stenosis as a complication of dermo necrosis was not found in the literature and, given the location and extent of the lesion, the absence of airway involvement was surprising, with only esophageal stenosis persisting for 2 years. The diagnosis of loxoscelism is usually late and the evolution is rapid and catastrophic. This case report demonstrates the importance of early suspicion and the severity of visceral and systemic complications. Continuing education for accident prevention, high suspicion and rigorous monitoring of systemic manifestations are necessary.

## CONFLICT OF INTEREST STATEMENT

The authors declare no conflicts of interest.

## ETHICS STATEMENT

All methods were performed by the relevant guidelines and regulations. The family involved (parent and/or legal guardian) signed the Informed Consent Form.

## Data Availability

The work data are arranged in an Excel spreadsheet, with the patient's full name. We will not provide this data for ethical reasons.
